# Assessment of the Root Canal Similarity in Contralateral Mandibular Incisors

**DOI:** 10.1016/j.identj.2022.04.003

**Published:** 2022-06-10

**Authors:** Gaute Floer Johnsen, Håvard Haugen, Liebert Parreiras Nogueira, Usame Sevgi, Ashley Mae Jimenez, Joseph T. DeLuca, Ryan Mancuso, Lucila Piasecki

**Affiliations:** a Department of Biomaterials, Institute of Clinical Dentistry, Faculty of Dentistry, University of Oslo, Oslo, Norway; b Oral Research Laboratory, Institute of Clinical Dentistry, Faculty of Dentistry, University of Oslo, Oslo, Norway; c Department of Periodontics & Endodontics, University at Buffalo, Buffalo, New York, USA

**Keywords:** Anatomic symmetry, Contralateral incisors, Micro-CT, 3D-matching, Imaging

## Abstract

**Introduction:**

The purpose of this study was to determine the degree of similarity between contralateral mandibular incisors utilising 3-dimensional (3D) models obtained from micro-computed tomographic (micro-CT) scans of extracted human teeth. The null hypothesis was that contralateral mandibular incisors do not exhibit matching symmetry.

**Methods:**

Sixty pairs (n = 120) of extracted mandibular incisors were obtained from 30 patients and scanned with micro-CT with a voxel size of 15.0 μm. 3D virtual models of the pulpal cavities were rendered. Geometric morphometric deviation analysis was performed after mirroring, automatic alignment, and co-registration of the models of contralateral teeth root mean square (RMS) errors were calculated. The quantitative analysis of the 3D models included 6 different geometric parameters. Data sets were examined with a 2-sample Kolmogorov–Smirnov test. Post hoc retrospective power analysis was performed to find statistical power (α = 0.05).

**Results:**

Contralateral pairs had a narrower distribution in deviation than random pairs. Also, contralateral pairs showed a statistically higher similarity coefficient (5 out of 6 geometric parameters) compared to random pairs (*P* < .001); no difference was found when comparing central to lateral pairs or between Vertucci type I configurations compared to non-type I. RMS errors had significantly lower Contralateral premolars (CPs) values than random pairs (*P* < .001).

**Conclusions:**

A high degree of similarity was demonstrated for pairing contralateral mandibular incisors using 3D models. The similarity between contralateral central and lateral incisors suggests that when screened and matched, these 4 teeth might be used in endodontic research where similar root canal anatomy is crucial.

## Introduction

For the dental operator to achieve a favourable outcome from root canal treatment (RCT), thorough knowledge and understanding of both the expected and the aberrant complex internal variations of root canal anatomy are paramount to successful shaping, debridement, and disinfecting the root canal system.^1^, ^2^, ^3^, ^4^

Several different destructive methods have been used for the study of the internal tooth anatomy of teeth. The development and application of 3-dimensional (3D) radiographic techniques in endodontic research, such as the noninvasive cone-beam computed tomography (CBCT) and nondestructive micro-computed tomography (micro-CT), have contributed to an increase in knowledge regarding root canal anatomy.^1^^,^^5^ Presently, micro-CT scans are considered the gold standard for investigating the internal anatomy of teeth.^5^

In 2016, Johnsen et al^6^ developed a methodology for the morphometric and geometric analysis of the anatomy of the pulp cavity by using 3D models of extracted teeth obtained by micro-CT scanning. Subsequent studies regarding the anatomy of contralateral premolars^7^^,^^8^ showed that teeth from the same patient present high similarity in many aspects of internal anatomy, except the apical third of the root canal. These findings highlight the importance of further investigation of variations in the internal anatomy of different types of teeth other than contralateral premolars.^8^

Mandibular incisors have one root that usually has a relatively simple canal configuration: a single long oval canal with one foramen.^2^ However, 2 or more canals have been reported with a prevalence ranging from 0.3%^9^ to as high as 50%.^10^ No studies have been performed to evaluate the similarity of contralateral mandibular incisors. Evaluating the similarities amongst teeth is of foremost importance since mandibular incisors have been extensively used to investigate the outcome of endodontic procedures and techniques.^11^, ^12^, ^13^, ^14^, ^15^ Moreover, recent studies have shown the importance of a comprehensive evaluation of the 3D anatomy when pairing teeth for research investigations.^16^^,^^17^

To the best of our knowledge, no previous studies have used 3D virtual models to compare the internal anatomy of contralateral mandibular central and lateral incisors. Thus, the aim of this ex vivo study was to determine the degree of similarity between contralateral mandibular incisors. The null hypothesis was that contralateral mandibular incisors do not exhibit matching symmetry.

## Materials and methods

### Sample selection

This study was approved by the local Institutional Review Board (no. 00003080), Department of Periodontics & Endodontics, University at Buffalo, New York, USA. One hundred sixty human mandibular incisors from 40 patients extracted for reasons not related to this study were selected. Teeth were visually inspected under magnification, and radiographs were taken from both clinical and proximal views. Teeth presenting with roots with cracks, calcified canals, immature apices, resorptive defects, extensive caries, filling material, or previous root canal access were excluded.

One hundred twenty sound mandibular incisors obtained from 30 patients were included in this study. The 4 incisors of each patient were inspected, identified, and mounted in a Styrofoam jig aligned as their respective arch position for easy identification. Samples were kept in plastic containers in 100% humidity.

### Nano-computed tomography and image processing

All specimens were scanned by a nano-computed tomography (nano-CT; SkyScan 2211 Multiscale X-ray Nano-CT System, Bruker micro-CT) with a 20- to 190-kV tungsten x-ray source and a dual detection system: an 11-megapixel cooled 4.032 × 2.670 pixel CCD-camera and a 3-megapixel 1920 × 1536 pixel CMOS flat panel. Contralateral mandibular incisors were scanned at 65 kV, 55 μA, and 120 ms. The scans were taken over 360° with a rotation step of 0.79° and a voxel size of 15.0 μm using the flat panel detector. This led to a scan duration of 13 minutes for each sample. Nano-CT projections were reconstructed using the system-provided software, NRecon (version 1.7.4.6), and analysed with CTAn (Bruker micro-CT, version 1.18.4.0). The resulting histogram was used to determine a binary threshold of 50 to 255 for the hard tissue.^18^

After scanning, the teeth were immediately transferred to a walk-in cold room maintained at constant 4 °C for storage in a 70% EtOH humidor. This was done in case there was a need for rescanning of teeth. The teeth were never in physical contact with the ethanol bath, and the evaporated ethanol was topped off regularly.

Bruker micro-CT software (CTAn, Bruker micro-CT, version 1.18.4.0) was used to extract the geometry and quantification of pulp data sets. A task list was created for the 3D geometric models to be treated as solids. It included analysis of several 3D parameters: object volume, surface area, surface over volume, object surface dentistry, surface convexity index, and structure index. The difference between the different contralateral pairs and randomly selected pairs where calculated. Random incisor pairs were acquired with an online randomiser.

Shape deviation analysis (*sda*) was done by geometric morphometric comparison with Geomagic Control® 2014 after alignment and co-registration as previously reported.^6^^,^^7^ Models were rendered superimposable with the “Mirror Model” function. The geometries were aligned in space by using automatic “Best Fit Alignment,” which uses an iterative closest point algorithm to align models^19^ with a tolerance of 0.00 µm. Next, fine-tuned automatic adjustments were made to the spatial position of 2 scans on the basis of all points in the 2 models through “Global Registration.” *Tolerance*, which specifies average deviation between points on different objects that, if reached, will stop the registration process, was set at 0.00 µm; *Maximum of Iterations*, which specifies the most iterations that will be performed whilst attempting to reach the registration goal, was set to 1000; *Sample Size*, which specifies the number of points from each point object that will be used to guide the registration process, was left unchanged at 2000. The *sda* settings for the spectrum defined 0.00 µm as maximum and minimum nominal values. The critical angle, the maximum difference between the normal of 2 points that suggest they lie on different faces, was set to 45°. The 3D comparison yielded both positive and negative deviations between the reference and test object and root mean square (RMS) errors. The comparison was performed for central and lateral pairs, different configurations, as well as randomised pairs.

### Statistical analysis

Data were analysed using the software package Graphpad Prism (GraphPad Software). Significance levels were set to *.05, **.01, and ***.001. The ROUT method with Q set to 1% was used to identify outliers as previously described,^20^ and 4 pairs were removed from the analysis.

All data sets were tested for normality and equality (Shapiro–Wilk). Since all data sets failed normality, the Kolmogorov–Smirnov test was performed to compare the different groups. Unpaired *t* test was used to compare 2 pairs to assess whether their population median ranks differ. All data sets were presented in box plots. A nonlinear multiple regression with goodness-of-fit analysis was performed on deviation distributions.

## Results

**Fig. 1:**
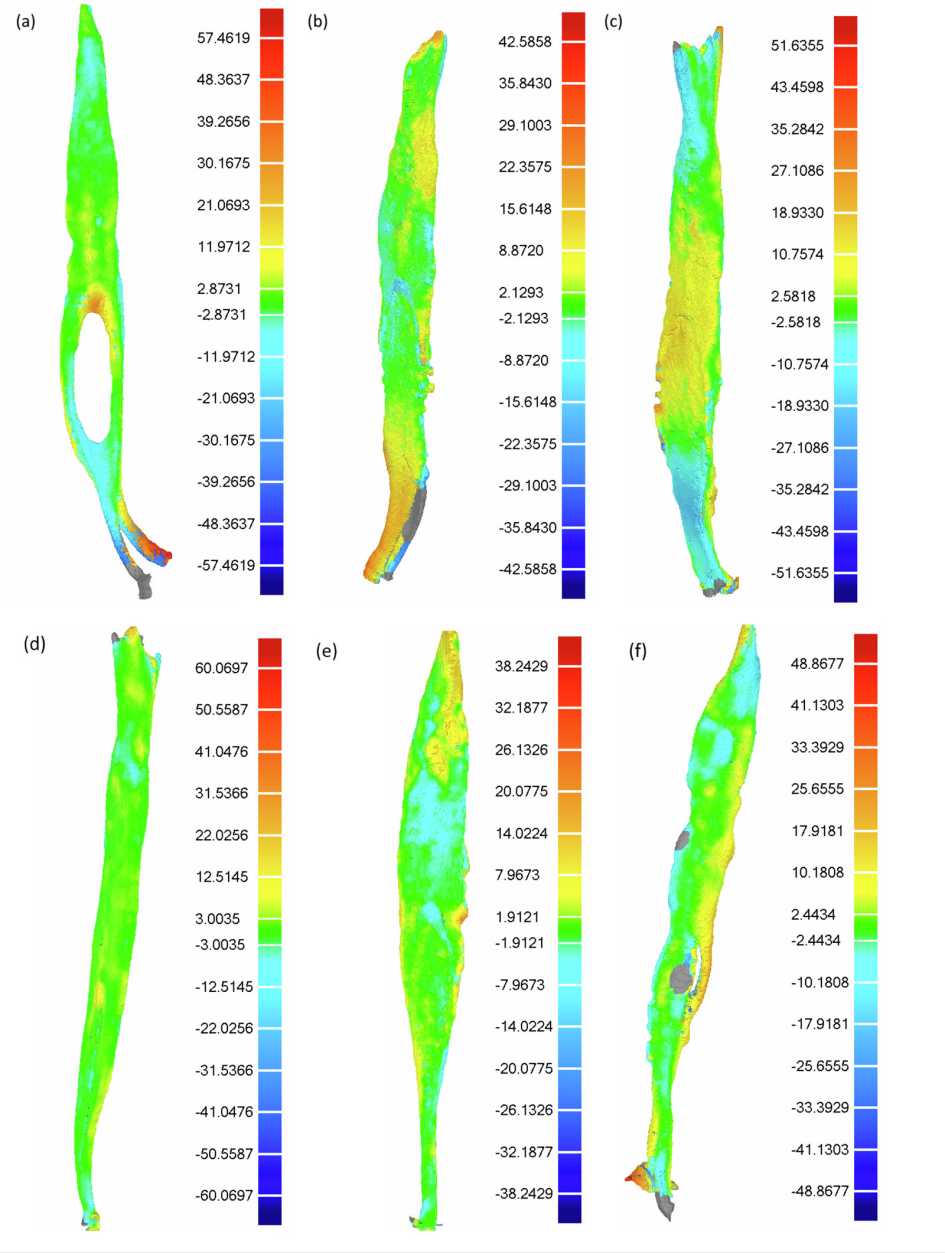
Each graph (a–f) represents the deviation map for a pair of contralateral incisors after mirroring and superimposition. The color-coded scale shows both complicated and straightforward anatomies with high degrees of similarity. Grey areas represent outliers beyond the maximum and minimum critical values of ±800.00 μm.

Four samples were removed because of alterations noted in the micro-CT scans such as cracks, fractures, or calcifications. Three-dimensional color deviation maps and a gradient key with 15 color segments from maximum to minimum critical values, with an exact match being set at 0.00 μm for nominal maximum and minimum values, were generated for contralateral incisors pairs (Figure 1).

**Fig. 2:**
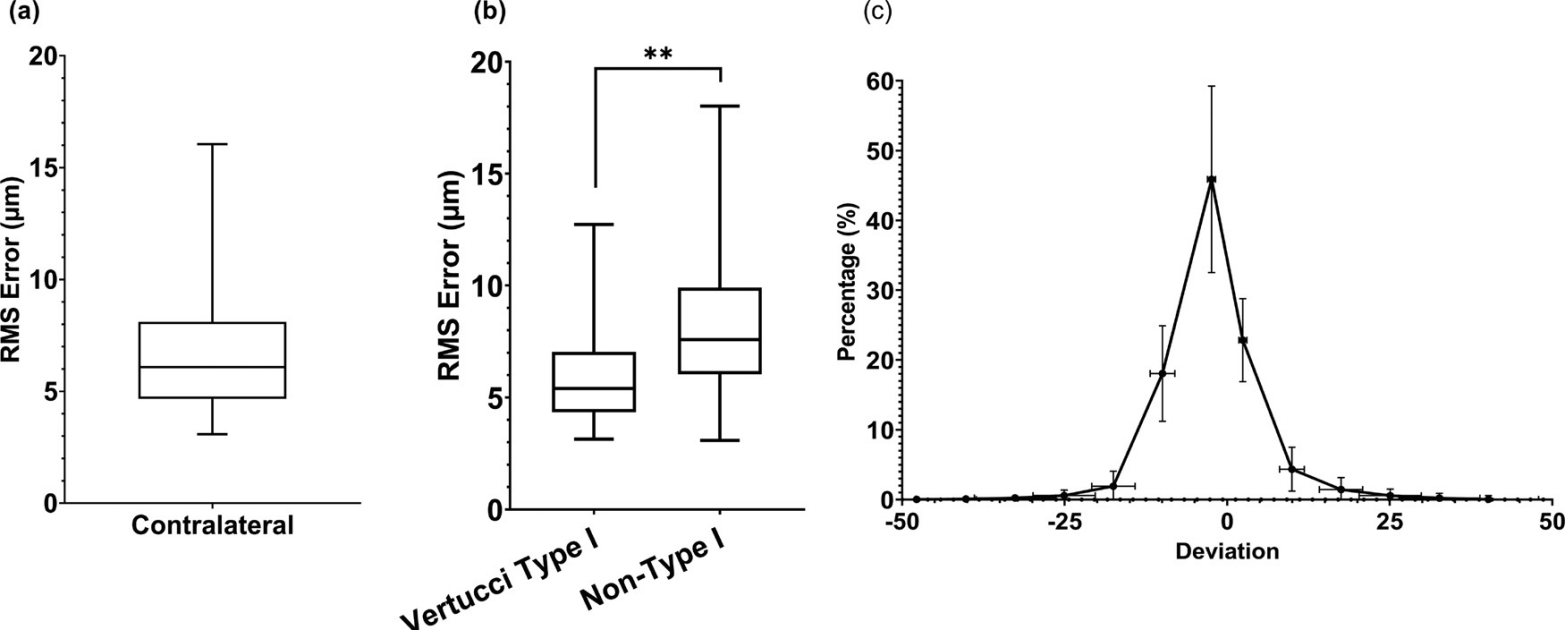
Root mean square error box plots of the paired contralateral incisors for all configurations (a) and split into Vertucci type I and non-type I configurations (b). The data are presented as median with 5th/95th percentiles along with descriptive parameters coefficient of variation, skewness, and kurtosis. The deviation distribution in geometries for all contralateral pairs, median values of all deviation profiles (c).

Deviation profile for all 56 contralateral incisors pairs and the average value of all pairs were plotted (Figure 2, c) along with standard deviations. The deviation profile for contralateral incisors pairs showed a deficient degree of deviation with a median of 0.9 and a high degree of similarity as the median goodness of fit was 0.994 ± 0.022 presented by the descriptive statistics.

**Fig. 3:**
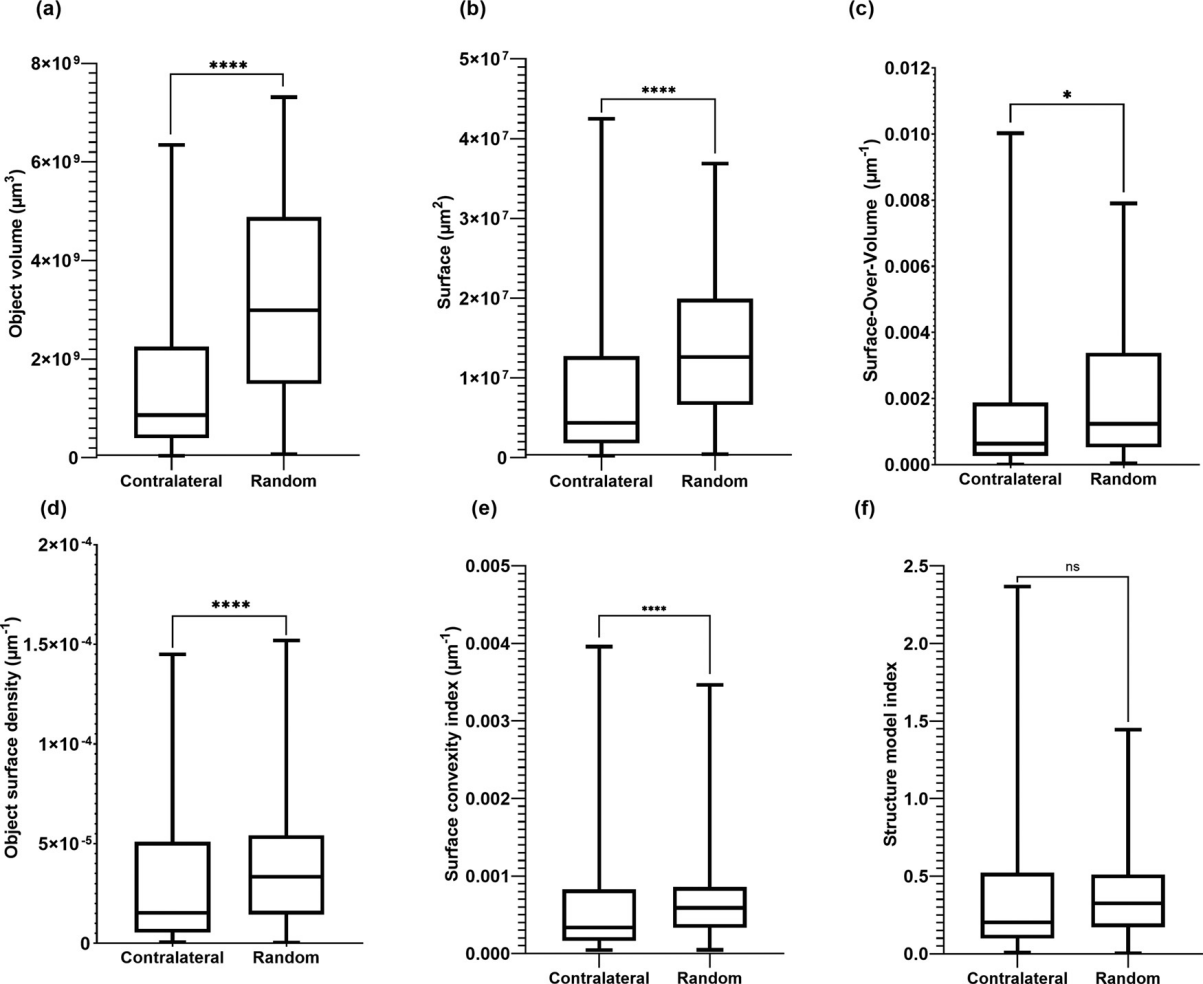
Box plots of 3D parameters comparing contralateral pairs and random pairs for (a) object volume, (b) surface, (c) surface over volume, (d) object surface density, (e) surface convexity index, and (f) structure model index. The data are presented as median with 5th/95th percentiles. ns = nonsignificant. *P < .05; ****P < .000.

The degree of similarity between contralateral incisors pairs was high, with a median RMS error value of 6.1; the deviation between the pairs was significantly lower for Vertucci type I configurations (5.4) vs non-type 1 configurations (7.6; *P* < .01; Figure 2, a and b). Both data sets have *P* < .0001 when tested in unpaired *t* test. Comparison of RMS errors for randomly selected pairs was performed as described by Johnsen et al^8^ (Figure 3).

**Fig. 4:**
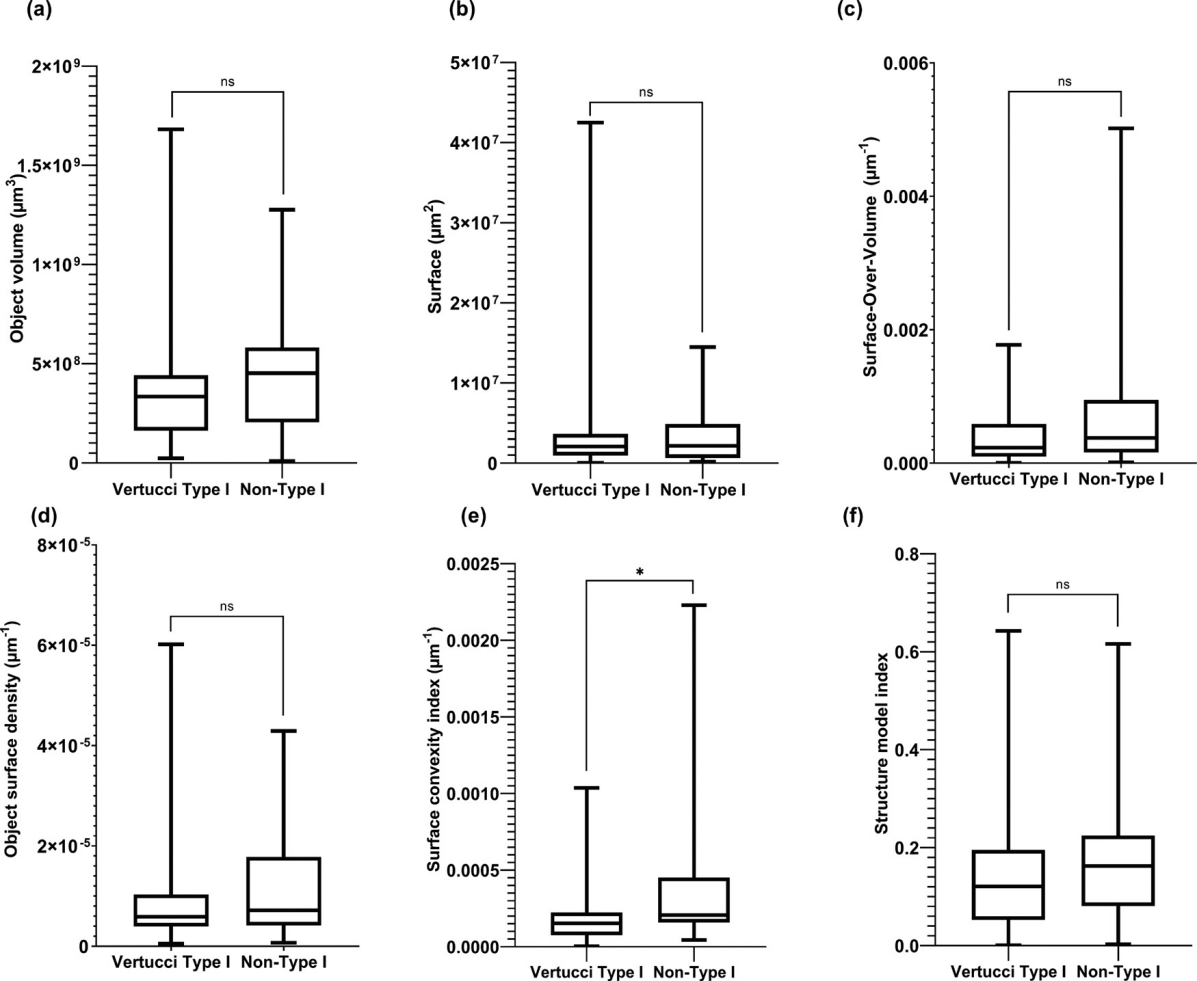
Box plots of 3D parameters comparing Vertucci type I to non-type I configurations for (a) object volume, (b) surface, (c) surface over volume, (d) object surface density, (e) surface convexity index, and (f) structure model index. The data are presented as median with 5th/95th percentiles. ns = nonsignificant. *P < .05.

Out of the 6 geometric parameters evaluated, the comparison between contralateral pairs and randomly selected pairs showed a high degree of significance difference (*P* < .0001) for object volume, surface, object surface density, and surface convexity (Figure 3, a–e) and a lower significance level (*P* < .05) for surface over volume; no significant difference was found for structure model index (*P* = .23). All data sets have *P* < .0001 when tested in unpaired *t* test. When comparing Vertucci type I to non-type I configurations, a significant difference (*P* < .05) was found only for surface convexity index (Figure 4). Similarly, no significance was found in comparing the same geometric parameters between central and lateral incisors.

## Discussion

The present study provides proof to the supposition that contralateral incisors are ideal as the substrate for root canal comparison studies based on the similarity between geometric and morphometric descriptors. Furthermore, this is the first study to include morphometric analysis and comparison of lateral and central incisor pulp space using micro-CT and semiautomated 3D software.

Although anterior teeth usually present a single root and canal, mandibular incisors have been reported to present more variable anatomy with a higher incidence of extra canals.^21^, ^22^, ^23^, ^24^, ^25^ A recent study evaluated 2543 CBCT scans in a Brazilian population and reported prevalence of 2 canals in 20% of mandibular incisors and bilateral symmetry on the root canal configuration close to 90%.^21^ On the other hand, a CBCT study in an Italian population showed that 2 canals occurred on average in 44% of the cases, whilst the symmetry was close to 45%.^26^ A CBCT study involving 1208 mandibular incisors from a sample obtained in Germany showed 2 root canals in 22.6% of patients and lateral incisors in 24.3% of patients.^27^ Interestingly, they found a high symmetrical occurrence of one root canal (72% in central and 76% in lateral incisors), but only symmetry in 16.6% (central incisors) and 19.2% (lateral incisors) in teeth with Vertucci type II configuration. Furthermore, symmetry seemed to decline with an increase in the complexity of configurations.

The internal anatomy of human teeth might be influenced by many factors related to the population, such as ethnicity, sex, and age. For example, it is well known known that the methods used for the investigation also play an essential role.^28^ Despite the 3D nature of CBCT scans, this method presents limitations in detecting complex anatomic configurations,^28^ explaining the contrasting results shown in the literature. Compared to micro-CT, CBCT has a larger field of view^29^^,^^30^ and larger voxel sizes.^24^^,^^28^^,^^31^, ^32^, ^33^, ^34^, ^35^ To date, most studies that have investigated the symmetry of mandibular anterior teeth using CBCT^26^^,^^36^, ^37^, ^38^, ^39^ lack a more detailed quantitative and qualitative analysis; the evaluations made were dependent on the observer, the comparisons were mainly made by classifying their Vertucci types or number of root canals and, because of limitations such as low resolution and slice thickness, more detailed analysis and in silico methods were not used for mirroring the anatomy of the contralateral teeth.

Micro-CT is considered the gold standard for studying dental hard tissues, but it is a time-consuming and costly methodology limited to smaller samples of extracted teeth. Moreover, studies using extracted teeth are usually performed using anonymised samples, lacking additional information that could be helpful for the proper identification of the teeth. This issue has foremost importance when studying mandibular incisors since central and lateral incisors present very similar morphology.

To overcome these limitations in the present study, only the samples containing 4 extracted mandibular incisors obtained from the same patient were included. This selection allowed for easy and efficient identification of the contralateral pairs. Additionally, teeth presenting with caries and/or restorations were excluded, preventing bias related to the alterations in the pulp cavity related to the deposition of tertiary dentin. However, because the age of the patients is unknown, one limitation of the present study can be related to the deposition of secondary dentine, which affects the root canal morphology as an individual ages.^40^, ^41^, ^42^

The calculation of RMS error has previously been shown as a vital matching error criterion in a 3D-matching study^43^ due to its indirect correlation with the magnitude of deviation.^6^ Furthermore, Johnsen et al have previously reported the similarity of contralateral premolar pairs using Geomagic Control (3D Systems GmbH) for an iterative closest point approach to achieve both alignments of the models in space and co-registration, followed by 3D models deviation analysis, thus providing quantitative data on morphometric characteristics independent from landmark placement.^6^^,^^8^ Here we used the same methodology for superimposing geometries, voxel registration used besides surface and landmark-based registration process to compare the difference of the geometries, with a high degree of accuracy. However, we observed a much higher degree of similarity than randomised pairs, which means that the internal pulp space and a root canal for contralateral incisors pairs have a higher degree of similarity when compared to premolar contralateral pairs. As such, they can be considered as mirror images of each other as well.

There is no study that has separately investigated the symmetry of mandibular incisors in terms of their Vertucci types. Nevertheless, the knowledge of the symmetry of the anatomic features of root canals is clinically significant and should be taken under consideration when treating contralateral teeth in the same patient.^37^ Despite the lower similarity observed for the non-type I sample than those with Vertucci type I, especially with the surface convexity index, they have been found to have a significantly more similarity with each other than randomly selected pairs.

It is known that the central and lateral mandibular incisors are very similar in their root canal systems and access cavities.^44^ In a micro-CT study, Leoni et al^10^ compared 50 central and 50 lateral mandibular incisors in 2- and 3-dimensional parameters regarding their volume, surface area, and structure model index, and the pairs showed no differences even when the samples were collected from different individuals. Our study supports these findings by using additional parameters in the 3D analysis, reporting no differences found between central and lateral pairs, and suggests that mandibular central and lateral pairs are interchangeable in their root canal anatomy.

Unfortunately, the outcome of canal preparation might be adversely affected by the fact that the anatomy of the root canal is highly variable.^45^ Therefore, information regarding anatomic complexities such as number of canals, shapes, trajectories, presence of presence of extra canals, bifurcations, and other irregularities should be considered when planning an endodontic treatment.^29^ This present study provides comprehensive information to the existing literature concerning similarity and symmetry of mandibular incisors.

The increasing knowledge about the similarity and symmetry of certain contralateral tooth types will help researchers to perform ex vivo studies in a more reliable, standardised, and practical way. Furthermore, the present study provides a standard for sample selection and standardisation for the endodontic comparison studies of aspects ranging from the effectiveness of instrumentation and irrigation techniques to root canal preparation. However, further studies are needed to validate the usage of the contralateral mandibular incisors as the standard tooth pairs in ex vivo comparative studies (Table 1 ).

**Table 1:** Descriptive for the root mean square error of the paired contralateral incisors for all configurations and split into Vertucci type I and non-type I configurations.

		Configuration	
	All	Vertucci type I	Non-type I
**Minimum**	3.1	3.1	3.1
**Median**	6.1	5.4	7.6⁎⁎
**Maximum**	16.1	12.7	18.0
**Coefficient of variation (%)**	43.5	37.8	50.7⁎⁎
**Skewness**	1.3	1.3	1.2
**Kurtosis**	1.6	1.7	0.7⁎⁎

⁎⁎ *P* < .001.

## Conclusions

A high degree of similarity was demonstrated for contralateral mandibular incisors using advanced computer algorithms along with metrology software. The similarity between central and lateral incisors implies that all 4 teeth, when screened and matched, can be used in endodontic research where identical root canal anatomy is crucial.

## Statement of authorship

Per the criteria defined by the International Committee for Medical Journal Editors (ICJME), please note the contribution made by each author listed in the manuscript. The submitting author affirms that all individuals listed as authors agree that they have met the criteria of authorship and agree to the conclusions of the study.

## Conflict of interest

None disclosed.
